# Mechanisms of object recognition: what we have learned from pigeons

**DOI:** 10.3389/fncir.2014.00122

**Published:** 2014-10-13

**Authors:** Fabian A. Soto, Edward A. Wasserman

**Affiliations:** ^1^Department of Psychological and Brain Sciences, University of CaliforniaSanta Barbara, Santa Barbara, CA, USA; ^2^Department of Psychology, University of IowaIowa City, IA, USA

**Keywords:** object recognition, categorization, invariance, learning, pigeon

## Abstract

Behavioral studies of object recognition in pigeons have been conducted for 50 years, yielding a large body of data. Recent work has been directed toward synthesizing this evidence and understanding the visual, associative, and cognitive mechanisms that are involved. The outcome is that pigeons are likely to be the non-primate species for which the computational mechanisms of object recognition are best understood. Here, we review this research and suggest that a core set of mechanisms for object recognition might be present in all vertebrates, including pigeons and people, making pigeons an excellent candidate model to study the neural mechanisms of object recognition. Behavioral and computational evidence suggests that error-driven learning participates in object category learning by pigeons and people, and recent neuroscientific research suggests that the basal ganglia, which are homologous in these species, may implement error-driven learning of stimulus-response associations. Furthermore, learning of abstract category representations can be observed in pigeons and other vertebrates. Finally, there is evidence that feedforward visual processing, a central mechanism in models of object recognition in the primate ventral stream, plays a role in object recognition by pigeons. We also highlight differences between pigeons and people in object recognition abilities, and propose candidate adaptive specializations which may explain them, such as holistic face processing and rule-based category learning in primates. From a modern comparative perspective, such specializations are to be expected regardless of the model species under study. The fact that we have a good idea of which aspects of object recognition differ in people and pigeons should be seen as an advantage over other animal models. From this perspective, we suggest that there is much to learn about human object recognition from studying the “simple” brains of pigeons.

Visually recognizing objects in the environment has a clear advantage for the survival and reproduction of any organism. Among many functions, it allows an animal to respond adaptively to sources of food, conspecifics, and possible threats. Although object recognition poses difficult computational problems (Rust and Stocker, 2010), humans and animals alike learn to respond similarly to nonidentical objects from the same category (categorization) as well as to respond differently to individual objects from the same category (identification).

Primates possess what are believed to be the most sophisticated visual systems among mammals. However, there is another vertebrate group that has also evolved highly advanced visual systems: birds (Shimizu and Bowers, 1999; Husband and Shimizu, 2001). For this reason, birds are the non-primate group in which high-level vision has been the most studied, and the pigeon is the species chosen in the majority of such studies (for reviews, see Cook, 2001; Wasserman and Zentall, 2006; Lazareva et al., 2012). This research has demonstrated impressive visual capabilities in pigeons, including the ability to detect and categorize many different classes of objects in a variety of conditions.

Object categorization and recognition have been studied in pigeons for 50 years, resulting in the accumulation of a large body of behavioral data (for previous reviews, see Huber, 2001; Kirkpatrick, 2001; Lazareva and Wasserman, 2008; Zentall et al., 2008). This accumulated knowledge affords us a unique opportunity for studying mechanisms of visual categorization that might be common to all amniote vertebrates (birds, reptiles, and mammals), which share a common evolutionary ancestor and basic organizational properties of their visual systems (Shimizu and Bowers, 1999; Husband and Shimizu, 2001; Shimizu, 2009). For these reasons, recent efforts in this line of research have been directed toward understanding the computational mechanisms that can explain the accumulated data. Here, we review the literature on object recognition and categorization in pigeons, with a special emphasis on the likely mechanisms involved, their plausible neurobiological substrates, and their evolution across vertebrates.

We will focus almost exclusively on object recognition and categorization. The large body of research on associative categories (i.e., stimulus equivalence; for a review, see Zentall et al., 2014) and artificial polymorphous categories (e.g., Lea et al., 2006) will be glanced here, and only in reference to related phenomena in object categorization. Furthermore, we will ignore categorization based on abstract stimulus properties, such as variability (Wasserman and Young, 2010), numerosity (Emmerton, 2001), relational properties (Vasconcelos, 2008), etc.

The review will be organized as follows. In section Behavioral Research on Object Categorization by Pigeons, we will review basic research on object categorization by pigeons. Because pigeons are assumed to have little or no experience with the objects presented to them in categorization experiments, an important part of this research has focused on object category *learning* instead of visual object *representation*, which is different from the focus of most human research (Soto and Wasserman, 2012b). Much like research in the area of perceptual learning in people (Lu et al., 2011), the evidence suggests that the learning of object categories by pigeons might result from the enhancement of selective readout from visual areas at a post-visual level, rather than from the direct modification of visual representations. Thus, a full account of what we know about object categorization in pigeons cannot focus exclusively on vision; we will review the learning mechanisms that might operate in non-visual areas of the pigeon brain in sections The Role of Error-driven Reinforcement Learning and Learning of Abstract Category Representations.

In section Visual Object Representation, we will turn to studies that have more directly assessed visual object representation in pigeons. We will show that many aspects of this research can be explained by feedforward processing of shape information, as implemented in models of primate vision.

In section The Evolution of Mechanisms of Object Recognition in Vertebrates: A Working Hypothesis, we will propose our current working hypothesis regarding the evolution of object recognition mechanisms in vertebrates, aiming toward explaining similarities and differences between pigeons and people (and other primates) found in behavioral studies. Finally, we argue in section The Neurobiological Mechanisms of Object Recognition: What We *Can* Learn From Pigeons that the pigeon could and should be used as an animal model of *some* of the computational processes involved in object recognition by people.

## Behavioral research on object categorization by pigeons

### Basic tasks and results

**Figure 1:**
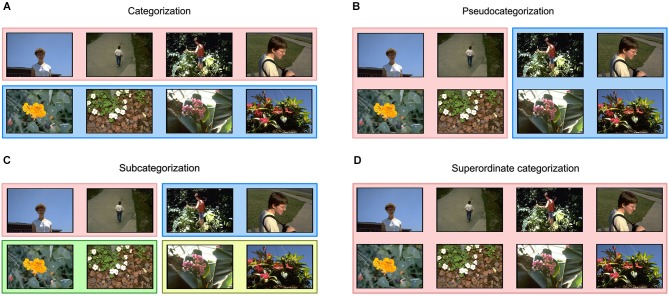
**Tasks commonly used in the study of object categorization by pigeons: Categorization (A), pseudocategorization (B), subcategorization (C), and superordinate categorization (D)**. Panels of different colors represent different responses assigned to the enclosed images.

Two basic tasks have been used to study object categorization by pigeons. Early research used go/no-go tasks, in which a single response is rewarded in the presence of some stimuli (go trials), but not in the presence of other stimuli (no-go trials). In the first published study in this area, by Herrnstein and Loveland (1964), pigeons were rewarded after pecking at a response key when a photograph included people, but they were not rewarded for responses to photographs without people. More recent research has used forced-choice tasks (see Figure 1A), in which several responses are made available at the same time (introduced by Bhatt et al., 1988). Pigeons are rewarded only when they peck at the response key assigned to the presented stimulus.

A large number of studies using both of these tasks have shown that pigeons can learn to categorize objects through feedback and, more importantly, pigeons can generalize discriminative performance to novel objects never seen before. The typical pattern of results is high performance with novel objects, but at a slightly lower level of accuracy than with the original training objects.

Pigeons are capable of learning categories comprising natural objects (Herrnstein and Loveland, 1964; Herrnstein et al., 1976; Herrnstein and De Villiers, 1980; Bhatt et al., 1988; Aust and Huber, 2001, 2002) human-made objects (Bhatt et al., 1988; Wasserman et al., 1988; Lazareva et al., 2004, 2006), scene gist (Kirkpatrick et al., 2014), cartoons (Matsukawa et al., 2004), human face identity (Soto and Wasserman, 2011), gender (Troje et al., 1999; Huber et al., 2000) and emotional expression (Jitsumori and Yoshihara, 1997), and even paintings from different artists (Watanabe et al., 1995; Watanabe, 2001).

The fact that pigeons can accurately classify new objects from known categories suggests that their brains can extract visual properties which are invariant across diverse members of such object categories. However, the information that the pigeon visual system extracts from images is even richer, allowing them to flexibly categorize the same images at different levels. For example, pigeons can learn *pseudocategorization* tasks (Figure 1B), in which photographs containing objects from several categories are randomly assigned to different sets (Herrnstein and De Villiers, 1980; Wasserman et al., 1988). Focusing on category-relevant visual information would actually hinder performance in pseudocategorization tasks. Thus, the birds must be capable of extracting many different object properties from photographs, some of them invariant across members of the category and others specific to a particular object.

In line with this idea, studies that have directly manipulated object properties in photographs have found that many features simultaneously control pigeons’ performance in a categorization task (e.g., Huber et al., 2000; Aust and Huber, 2002; Lea et al., 2013), with variations in performance being well explained as a linear function of the presence or absence of such features (Huber and Lenz, 1993; Jitsumori and Yoshihara, 1997).

**Figure 2:**
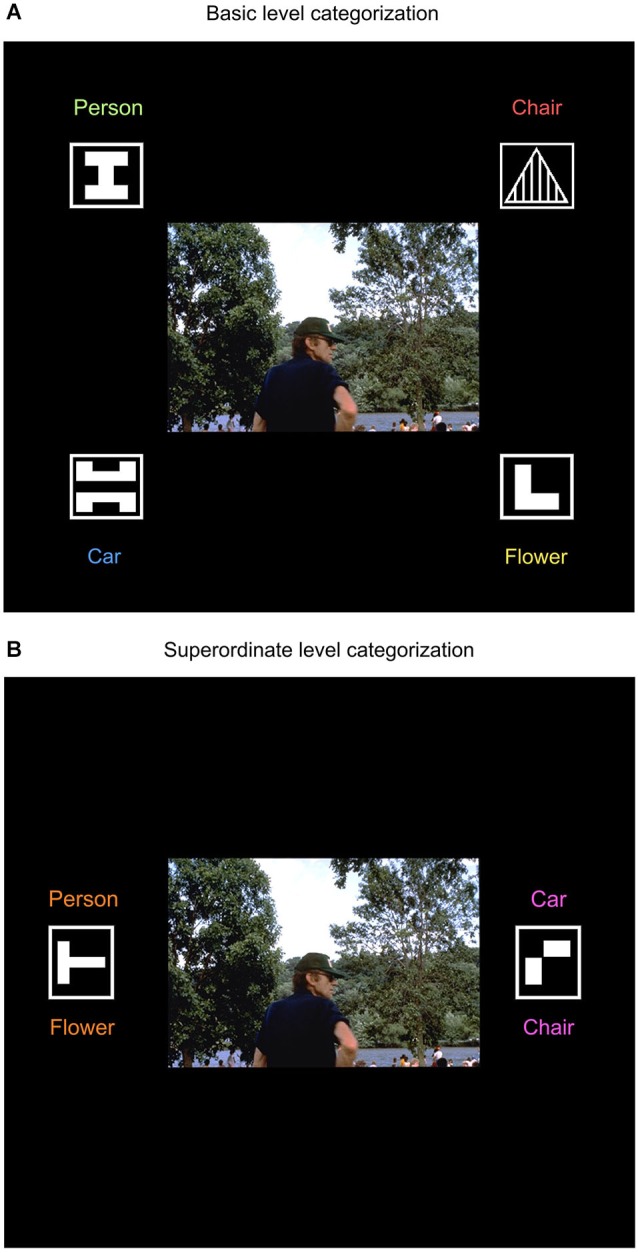
**Schematic layout of a basic-level categorization trial (A) and a superordinate-level categorization trial (B) in experiments studying pigeons’ ability to flexibly categorize the same object at different hierarchical levels**.

An important aspect of human categorization is that the same object can be flexibly categorized at several different hierarchical levels. For example, the photograph of a human can be categorized at the so-called “basic” level as a person, at the “superordinate” level as an animal, and at the “subordinate” level as “John”. Pigeons, too, have shown the ability to flexibly categorize the same objects (cars, chairs, flowers, and people) at different levels, depending on task demands (Figure 1D; Lazareva et al., 2004). The procedure used to train such flexible categories is illustrated in Figure 2. When the photograph of a human is presented together with four response keys, the pigeons learn to classify it at the basic level (Figure 2A), whereas when the photograph is presented together with two different response keys, the pigeons learn to classify it at the superordinate level of “natural object” (Figure 2B), comprising both people and flowers.

The success of pigeons in the task shown in Figure 2 is evidence for the flexibility in their categorization skills. However, it could be argued that learning to give the same response to two object categories is a far cry from forming a common superordinate representation for them. Other evidence shows that pigeons do in fact learn common superordinate representations in this type of task. For example, when objects from two perceptually dissimilar categories are associated with the same response, new learning obtained with objects from one of the categories automatically transfers to objects from the other category (Wasserman et al., 1992; Astley and Wasserman, 1998, 1999). This transfer suggests that training with a common response leads to the emergence of a single representation for both categories, which then mediates new learning about either of them. Such learning of a common representation for all stimuli associated with the same response is not restricted to superordinate categories, as it can be found after training with basic categories (Vaughan and Herrnstein, 1987) and with pseudocategories composed of two or more perceptually-dissimilar stimuli (Vaughan, 1988). This learning phenomenon, named stimulus equivalence in the behavioral literature (for a review, see Zentall et al., 2014), can also be found when members of a category share a common association with a particular stimulus or reward, instead of with a specific response.

In summary, the basic features of object category learning in pigeons are the following. First, pigeons can learn a variety of complex object categories and transfer this learning to novel objects. Second, pigeons can flexibly classify the same object according to different criteria (e.g., pseudocategories and superordinate categories). Third, pigeons extract a rich variety of visual properties from photographic images and use them in combination to learn the structure of object categories. Finally, pigeons learn common abstract representations for all members of the same trained category.

### Variables that affect object category learning

Several factors affect both the speed with which pigeons learn new object categories and the level to which they can generalize this knowledge to unseen objects. One of the factors that has a strong effect on object categorization by pigeons is the similarity relations between objects in the same category (within-category similarity) and between objects in different categories (between-category similarity) included in the same training task. It is generally believed that natural basic object categories have a higher level of within-category similarity than between-category similarity, what is termed “perceptual coherence”. For this reason, several early studies sought evidence as to whether pigeons could perceive and use such perceptual coherence for categorization, in contrast to just learning object categories by rote memorization of the images.

For example, Astley and Wasserman (1992) rewarded pigeons for pecking at photographs from a target category and measured to what extent the pecking response generalized to non-rewarded test objects. Some of these test objects belonged to the target category and others belonged to different categories. Higher responding to objects from the target category would be a indication that pigeons perceive within-category similarity as being higher than between-category similarity. Such categorical generalization was high early in the experiment, but slowly fell as pigeons acquired experience with non-rewarded presentations of the test stimuli.

Several pieces of evidence suggest that the perceptual coherence of object categories biases pigeons to group objects together into basic categories, even when this categorical bias goes against the prevailing task demands and is therefore costly in terms of earned food reward. One example comes from experiments comparing the learning of real categories and pseudocategories (Figures 1A,B). When the perceptual coherence of categories is eliminated by randomly assigning objects to pseudocategories, learning of the task slows down compared to when perceptual coherence is maintained (Herrnstein and De Villiers, 1980; Wasserman et al., 1988).

**Figure 3:**
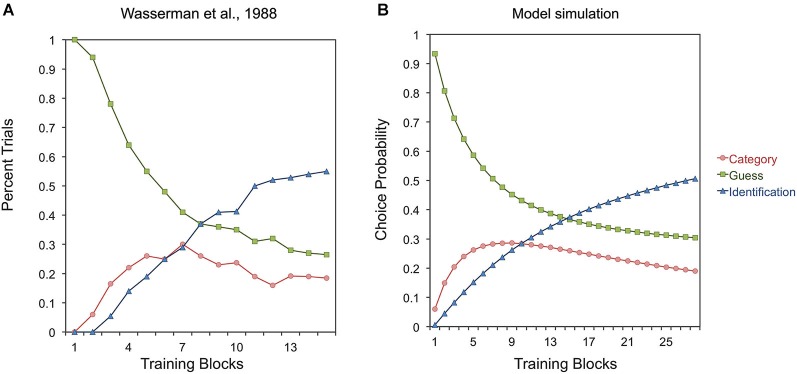
**Experimental results (A) and simulated results (B) of a study on the strategies used by pigeons at different stages of learning a subcategorization task (Wasserman et al., 1988)**. The “Categorization”, “Identification” and “Guess” series represent the proportion of trials in which pigeons and the model used categorization, identification or random guessing as a response strategy, respectively.

A categorical bias is also clearly observable in “subcategorization” tasks, in which two different responses are assigned to objects from the same category. In one experiment (Wasserman et al., 1988), illustrated in Figure 1C, objects from one category were assigned to two separate response keys, and objects from a second category were assigned to two other response keys. In this task, if the pigeons randomly choose a response key, then they get 25% correct responses. Pigeons can also learn about which two response keys are associated with each category, in which case they get 50% correct responses, but 50% categorical errors. Thus, this categorization strategy leads to above-chance performance, but it is not the strategy leading to the best payoff. The optimal strategy is learning to identify each individual stimulus and its correct response. When Wasserman et al. estimated the percentage of trials in which the pigeons were following each strategy, they found the results shown in Figure 3A. Although it is not the best strategy, pigeons first learn to categorize stimuli, and only later learn to identify them.

The categorical strategy shown by pigeons in the early blocks in Figure 3A is not optimal, but it does produce better reward payoff than guessing. Soto and Wasserman (2010b; see also Soto et al., 2012) found that a similar categorical bias can be found using a go/no-go subcategorization task. In this task, responses to a group of objects never produce reward, yet early in training pigeons respond to them at the same level as to rewarded objects from the same category. That is, pigeons learn first to categorize objects in subcategorization tasks, regardless of whether or not this strategy produces reward.

The previous experiments all suggest that pigeons perceive the within-category similarity of objects in natural photographs to be higher than their between-category similarity. This result is not trivial; it is important that the category structures that pigeons are biased to learn are exactly those that are likely to be encountered in the natural environment (see Smith et al., 2010). However, even when they are learning artificial categories, pigeons (Cook and Smith, 2006) and primates (Blair and Homa, 2003; Smith et al., 2010) show a bias to learn perceptually-coherent category representations before learning information about specific stimuli.

Differences in between-category similarity also play a role in category learning. For example, Aust and Huber (2002) concluded that how much responding to the trained category “person” generalized to similar or related categories (such as dolls, primates, mammals, and birds) depended on how many features were shared by the categories.

When pigeons are concurrently trained to classify the same categories at both basic and superordinate levels, it is usually found that they learn the basic task faster for some categories and the superordinate task faster for other categories (Lazareva et al., 2004, 2010; Lazareva and Wasserman, 2009). Lazareva et al. (2010) obtained estimates of the similarity among four object categories by analyzing generalization data through multidimensional scaling. Then, they showed that such similarity estimates could predict whether the basic or superordinate levels would show an advantage for different pairs of categories. A superordinate-level advantage is seen when the two categories in a superordinate set are perceptually similar, whereas a basic-level advantage is seen when the two categories in a superordinate set are perceptually dissimilar. This result is interesting because it is in line with one of the hypotheses put forward to explain the basic-level advantage in humans (Rosch et al., 1976).

**Figure 4:**
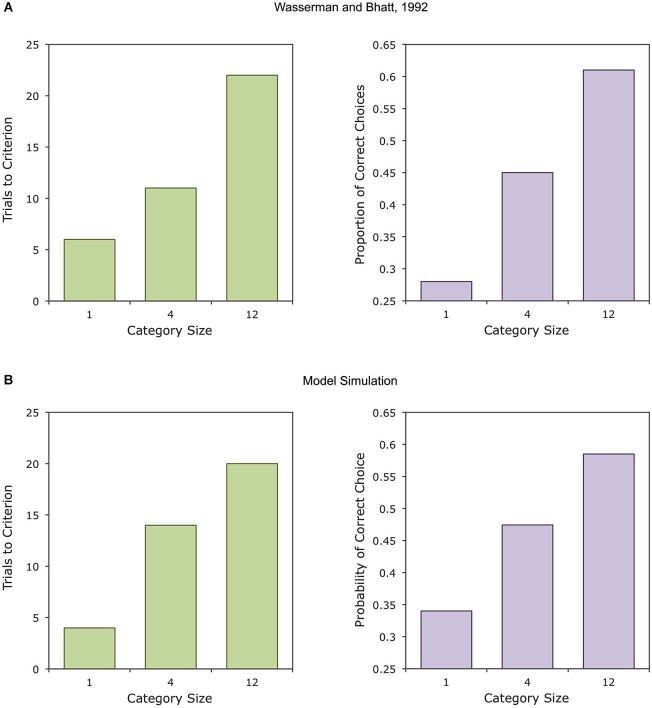
**Experimental results (A) and simulated results (B) of a study on the effect of category size on object category learning by pigeons (Wasserman and Bhatt, 1992)**. Category size increases the number of trials to reach a criterion of 0.7 proportion correct (left) and increases generalization to novel objects from the trained categories (right).

Factors related to the training regime also affect category learning and generalization. One such factor is the number of different objects in each category presented during training (Kendrick et al., 1990; Astley and Wasserman, 1992; Wasserman and Bhatt, 1992). As shown in Figure 4A, learning of the categorization task is slowed and transfer of performance to new images is enhanced with a higher number of training exemplars. In the extreme case, in which training images are never repeated, pigeons can still learn the object categories, but learning is slower than when the training exemplars are repeated (Bhatt et al., 1988).

**Figure 5:**
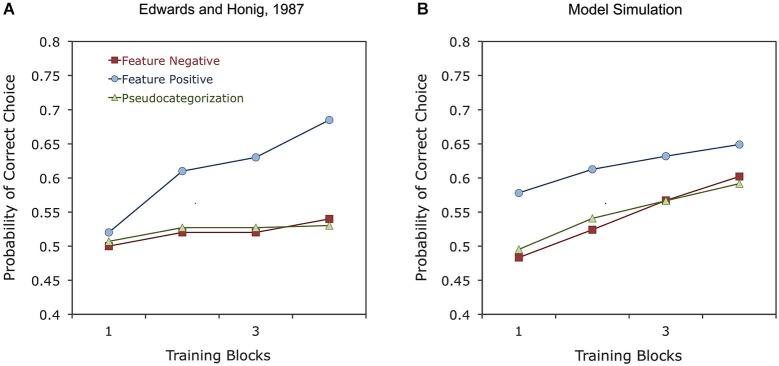
**Experimental results (A) and simulated results (B) of a study on the feature-positive effect in object category learning by pigeons (Edwards and Honig, 1987)**. In the feature-positive discrimination, objects from a category predict the delivery of reward, whereas in the feature-negative discrimination, objects from a category predict absence of reward. In the pseudocategorization task, different objects from the same category predict either reward or no reward.

Another important training factor for studies using a go/no-go task is whether responses are rewarded to images showing the category, in what is called a feature-positive task, or to images showing no category, in what is called a feature-negative task. For example, Edwards and Honig (1987; see also Aust and Huber, 2001, 2002) trained pigeons to discriminate photographs of various scenes from photographs of the same scenes with people in them. Their results, reproduced in Figure 5A, show that pigeons were quite fast in learning the feature-positive discrimination, in which responses to people were rewarded, but they were slow in learning the feature-negative discrimination, in which responses to scenes without people were rewarded. In fact, learning of the feature-negative discrimination was as slow as learning a pseudocategorization task, suggesting that pigeons do not show any benefit from perceptual coherence when responses to the category are not rewarded.

Patterns of generalization also vary for feature-positive and feature-negative tasks. Aust and Huber (2001) trained pigeons with the “people” category in feature-positive and feature-negative tasks. After training, pigeons were presented with new combinations of background scenes and people that involved contradictory information. For example, either familiar or novel people, which were associated with one outcome during training (e.g., reward), could be presented on familiar backgrounds, which were followed by the opposite outcome during training (e.g., no reward). The authors found that feature positive training led to generalization of the response learned for people to these conflicting test stimuli, whereas feature negative training led to no preference to respond or to inhibit responding to conflicting test stimuli. Again, this finding suggests that learning about the whole category is possible only when responses to the category are rewarded, but not when responses to the category are not rewarded.

## The role of error-driven reinforcement learning

What learning mechanisms could give rise to the features of object category learning we reviewed in the previous section? We have recently shown (Soto and Wasserman, 2010b, 2012c) that most of this research can be explained by a model implementing two simple assumptions. The first assumption is that objects from any category are represented by a large common collection of features or “elements”, with different categories involving different probabilities that an object from the category will activate each of those common elements. When the probability of activation of an element is high in a particular category, that element is activated by several different objects from that category, rendering it relatively *category-specific*. When the probability of activation of an element is low in a particular category, only a few objects from the category activate the element, rendering it relatively *stimulus-specific*.

The second assumption is that category learning proceeds by strengthening connections between such elemental representations and responses through error-driven learning. As in some reinforcement learning systems (Kaelbling et al., 1996; Sutton and Barto, 1998), on each trial, the model selects an action that is likely to maximize predicted reward, usually the action with the strongest connections to active elements. The difference between the predicted reward and the reward obtained after the response is made–reward prediction error–determines how much the connection between the active elements and the chosen action should be modified (Rescorla and Wagner, 1972).

Note that this model is deliberately abstract regarding object representation: the elements do not have specific semantic content (i.e., they do not represent specific features), they only play different roles depending on what information they carry about the category. Furthermore, specific object and category representations are irrelevant, as they are randomly sampled in each simulation and the results of many simulations are then averaged to generate predictions. This process allowed us to ignore many questions about visual representation, while testing to what extent our two simple assumptions can explain pigeons’ behavior. The resulting learning model is compatible with any account of visual processing which produces representations in line with our assumptions; indeed, we expanded the model in precisely this direction, as we will see below.

Our model specifies the conditions leading to the control of actions by category-specific elements, yielding categorization learning; it also specifies the conditions leading to the control of actions by stimulus-specific elements, yielding identification learning. For example, all instances of the categorical bias discussed in the previous section are the result of differences in the rate at which category-specific and stimulus-specific elements are presented in a typical categorization task. Because category-specific elements are shared by many objects, they are presented often and their connections with responses can be modified faster. Stimulus-specific elements are presented less often and they support slower learning. In short, category-specific elements have a repetition advantage over stimulus-specific elements.

As seen in Figure 3B, this repetition advantage can explain the reliance on a categorization strategy shown by pigeons during the early stages of learning in a subcategorization task (Wasserman et al., 1988; see Figure 3A). Early in training, category-specific elements quickly strengthen their connections with the two different responses with which a category is paired, producing above chance accuracy. However, this tendency also results in a large proportion of categorical errors due to within-category generalization. To reduce such categorical errors, the connections between stimulus-specific elements activated by particular objects and the incorrect response become inhibitory. This inhibitory learning is slow due to the low rate of presentation of stimulus-specific elements, but it eventually leads to better discrimination performance at the end of training by canceling generalized excitation from one subcategory to the other.

Note how strongly the repetition advantage effect depends on the number of objects included in the training set. With just one object, the effect does not occur because all types of elements are presented equally often. As the number of objects increases, category-specific elements are presented quite often (in the extreme, on each trial from the same category), whereas presentations of stimulus-specific elements become more and more rare (in the extreme, once for each object repetition). As shown in Figure 4B, this analysis explains the effect of category size on learning rate and generalization. With a small category size, the same elements are repeated on each trial and learning about a specific stimulus is fast. However, there is no repetition advantage effect for category-specific elements and generalization to new objects is poor, as it depends on control by such common elements. The opposite is true when category size is increased.

Some particular features of error-driven learning help explain other results. For example, the faster learning of feature-positive discriminations, reproduced by the model in Figure 5B, stems from the fact that such discriminations require the model to first learn to respond to a number of rewarded stimuli and then to inhibit generalized responding to non-rewarded stimuli. This two-stage process is a signature of error-driven learning: inhibitory learning does not occur without an excitatory context to provide negative prediction errors, so excitatory learning must occur first. In the feature-positive discrimination, the repetition of category-specific elements boosts excitatory learning at the beginning of training, whereas in the feature-negative discrimination, pigeons must first learn to respond to each individual background independently, which takes longer. For a detailed explanation of other feature-positive effects, as well as many other results from the literature, see Soto and Wasserman (2010b).

### Direct empirical evidence for error-driven learning

More recent experiments, motivated by the model described in the previous section, have led to more direct evidence for the role of error-driven learning in object categorization by pigeons. The important insight provided by the model is that different tasks can be used to manipulate the connections between different types of elements (category-specific and stimulus-specific) and responses.

**Figure 6:**
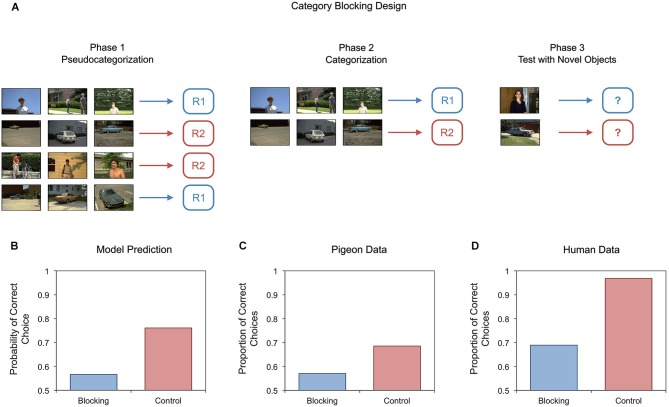
**Schematic diagram of an experiment on blocking of object category learning (A), together with our model’s predictions (B) and experimental results of studies with pigeons (C) and people (D)**. Bars in the bottom figures represent responding to novel test objects from the training categories during Phase 3.

One example is the blocking design illustrated in Figure 6A. In the blocking condition (Soto and Wasserman, 2010b,d), objects from the same category are first assigned to different responses in a pseudocategorization task (Phase 1). According to the model, accurate performance in this task requires strong connections between stimulus-specific elements and the correct responses. Once the pseudocategorization task is learned, it is possible to transform it into a true categorization task by dropping half of the trials, as shown in the middle panel of Figure 6A (Phase 2). Under normal circumstances, experience with this new categorization task should lead to strong control by category-specific elements and good generalization to new objects when they are presented during a test (Phase 3). In a control condition, pigeons were exposed only to this categorization task and a generalization test (Phases 2 and 3). In the blocking condition, however, the stimulus-response mapping is already known at the beginning of Phase 2; thus, pigeons should make few, if any, errors in predicting the correct response for each of the stimuli in this phase. No prediction error means no category learning; so, the model predicts less generalization of categorical performance to new objects in the blocking group than in the control group.

The predictions of the model and the performance of pigeons with novel objects in each condition are shown in Figures 6B,C, respectively. It can be seen that pigeons showed the predicted pattern of results. This blocking effect, analogous to effects found in Pavlovian conditioning (Kamin, 1969), is direct evidence that object category learning in pigeons is driven by reward prediction error.

The blocking effect also helps to explain some contradictory results in the literature. For example, Sutton and Roberts (2002) used a design very similar to that of Astley and Wasserman (1992) to study the “perceptual coherence” of object categories, but found that generalization was the same to objects from any category, not only the target category. We have shown (Soto and Wasserman, 2010b) that Sutton and Roberts’ results can be explained as a blocking effect, in which elements common to all of the object categories acquire control over performance early in training.

**Figure 7:**
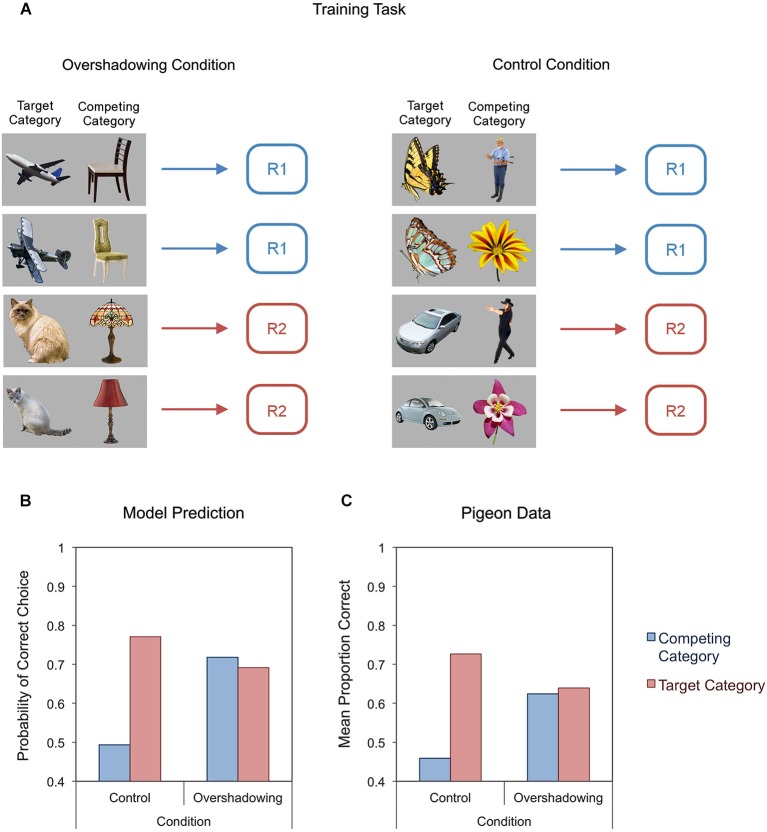
**Schematic diagram of an experiment on overshadowing of object category learning (A), together with our model’s predictions (B) and experimental results from an experiment with pigeons (C)**. Bars in the bottom figures represent responding to novel test objects from the training categories.

Other studies have found evidence of an *overshadowing* effect in category learning (Soto and Wasserman, 2012a; Soto et al., 2012). Figure 7A shows a schematic representation of the training tasks given to pigeons in one of these experiments (Soto and Wasserman, 2012a). On each trial, two different objects were presented to the pigeons. In the overshadowing condition, these objects came from two categories that were both informative about the correct response. For example, in Figure 7A, both airplanes and chairs were consistently associated with Response 1. Here, the category-specific elements of both categories should acquire control over behavior quite fast, quickly reaching a point in which performance is good and learning stops. At this point, the two categories overshadow each other: each acquires only a proportion of the response control that they would have gained if they had been presented alone. In the control condition, two objects are presented in each trial, but a single target category is informative about the correct response. In the example in Figure 7A, butterflies and cars are informative about correct responses, but people and flowers are not. In both conditions, category learning was tested by presenting pigeons with new objects from the trained categories. As shown in Figure 7B, the model predicts that performance with the target categories (red bars) should be impaired in the overshadowing condition compared to the control condition. As shown in Figure 7C, this prediction of the model matched the pigeons’ behavior. Furthermore, performance with the competing categories (blue bars) was also close to the model’s predictions.

### Prediction error as a general mechanism of object category learning

Given the accumulated evidence suggesting that error-driven learning plays an important role in object categorization by pigeons and the fact that this form of learning is widespread across species and tasks (Siegel and Allan, 1996; Bitterman, 2000; Macphail and Bolhuis, 2001), it seems likely that similar mechanisms underlie object categorization in primates, including humans.

A repetition advantage effect for category-specific properties seems to be as important in people as it is in pigeons. For example, the categorical bias effects and category size effects that are pervasive in the pigeon literature can also be found in people and other primates (Homa et al., 1973; Smith and Minda, 1998; Minda and Smith, 2001; Smith et al., 2010). As indicated earlier, such effects result naturally from the interaction of a repetition advantage for category-specific information and error-driven learning (see Soto and Wasserman, 2010b).

The results of behavioral experiments suggest that error-driven learning plays an important role in object categorization in people. Just as with pigeons, when people are trained to solve a discrimination task by memorizing individual objects in photographs and their assigned responses, they are impaired in detecting a change in the training circumstances in which all of the presented objects are sorted according to their basic-level categories (Soto and Wasserman, 2010d). That is, people show a category blocking effect, as illustrated in Figure 6D (see also Gluck and Bower, 1988; Shanks, 1991; Nosofsky et al., 1992).

We have proposed (Soto and Wasserman, 2012c) that underlying these behavioral similarities is an evolutionarily conserved learning mechanism that might be implemented in the basal ganglia, which are homologous structures in birds and mammals (Reiner, 2002; Reiner et al., 2005). Many studies implicate the basal ganglia in visual categorization and other visual discrimination tasks in people and other primates (Ashby and Ennis, 2006; Seger, 2008; Shohamy et al., 2008). The basal ganglia receive input from most sensory areas and send output to motor areas, which allows for the sensory integration and response selection functions necessary for category learning. The input nuclei in the basal ganglia, collectively known as striatum, receive dopaminergic input from the subtantia nigra pars compacta (Durstewitz et al., 1999; Nicola et al., 2000; Reiner et al., 2005) and the plasticity of cortical-striatal synapses depends on the presence of this dopaminergic input (Centonze et al., 2001; Reynolds and Wickens, 2002). As there is considerable evidence that the activity of these dopaminergic neurons is correlated with reward-prediction error (Montague et al., 1996; Schultz, 1998, 2002; Waelti et al., 2001; Suri, 2002), cortical-striatal synapses (pallial-striatal synapses in birds) may mediate the error-driven learning of associations between visual representations and responses. As proposed by our model, learning in the striatum during object categorization tasks would require activity of the presynaptic visual neurons (stimulus elements in the model), activity of the postsynaptic striatal neurons (actions in the model), and the presence of a dopaminergic signal (reward prediction error in the model).

## Learning of abstract category representations

Some of the features of object category learning in pigeons mentioned in section Basic Tasks and Results cannot be explained by the reinforcement learning account described in the previous section. In particular, a model that only learns associations between stimulus properties and responses cannot explain the vast behavioral evidence that pigeons (and other vertebrates) learn a common representation for all members of a category associated with the same response (“stimulus equivalence”; for a review, see Zentall et al., 2014).

Evidence from a neurophysiological study suggests that such a common representation may have a substrate in the nidopallium caudolaterale (NCL), where neurons can be found that respond similarly to perceptually dissimilar stimuli associated with a common response (Kirsch et al., 2009). These results were interpreted as indicating that categorization learning established category-selective coding of the stimuli in NCL, and they are similar to findings in the primate prefrontal cortex (PFC; Freedman et al., 2001, 2002, 2003).

Just as is the case of the primate PFC, the avian NCL receives massive dopaminergic projections from the midbrain (Wynne and Güntürkün, 1995; Durstewitz et al., 1999; Kröner and Güntürkün, 1999) as well as input from neurons in both visual and sensorimotor areas (Leutgeb et al., 1996; Kröner and Güntürkün, 1999). NCL is thus particularly well suited to integrate information from several different sensory modalities through dopamine-modulated learning.

This result is important because the observation that neurons in lateral PFC come to respond selectively to the category of a stimulus and other behaviorally relevant factors in an object categorization task (Freedman et al., 2001, 2002, 2003) has led to wide acceptance, among primate researchers, of the hypothesis that PFC is the most critical site for object category learning (Freedman et al., 2003; Jiang et al., 2007; Serre et al., 2007). One possibility is that primate PFC and avian NCL implement learning of a common abstract representation for objects belonging to the same category, whereas stimulus-response associative learning is implemented in the basal ganglia (Antzoulatos and Miller, 2011). This possibility could explain why the PFC does not seem to be necessary for performance and generalization of category learning in monkeys (Minamimoto et al., 2010).

## Visual object representation

In the previous sections, we reviewed a line of research in pigeons that focused on object category learning. A different, but related line of research in pigeons has been heavily influenced by the human literature on invariant object recognition. As in the human literature, this line of research has been strongly focused on questions about object representation, such as: Which object properties are important for object recognition in pigeons? Can pigeons extract invariant object representations? Can pigeons show invariant object recognition after limited experience with an object? The following two sections will focus on this literature.

### Invariance in object recognition by pigeons

Following the human literature, much research in object recognition by pigeons has focused on whether or not this species can show recognition that is invariant to changes in identity-preserving variables, such as rotation, scaling, illumination, etc. In general, the results of psychophysical experiments all point to the same conclusion: pigeons’ object recognition after training with a single object image is controlled by a variety of properties that are irrelevant to object identification. In order to show invariant object recognition, pigeons require training with variations in such irrelevant properties.

For example, experiments that have explored whether pigeons show view-invariant object recognition after being trained with only one object view have uniformly found significant costs of object rotation on accuracy, regardless of the type of object used to generate the experimental stimuli (Cerella, 1977; Lumsden, 1977; Wasserman et al., 1996; Peissig et al., 1999, 2000, 2002; Friedman et al., 2005). Similarly, other experiments have found that, after experience with a single image view, pigeons’ object recognition is affected by variations in size (Larsen and Bundesen, 1978; Pisacreta et al., 1984; Peissig et al., 2006), shading (Cabe, 1976; Cook et al., 1990; Young et al., 2001), and position (Kirkpatrick, 2001).

Although object recognition in people is far from being completely invariant (Jolicoeur, 1987; Hayward and Tarr, 1997; Tarr et al., 1998; Kravitz et al., 2008), it is clear that humans show greater invariance than do pigeons (Biederman and Ju, 1988; Biederman and Cooper, 1992; Biederman and Gerhardstein, 1993; Hayward, 1998). For example, people, but not pigeons, have been shown to exhibit view-invariant recognition when they are tested with the appropriate stimuli (Biederman and Gerhardstein, 1993) and show view-invariant recognition of novel views of an object which are interpolated between experienced views (Spetch and Friedman, 2003). Furthermore, some factors that are known to foster view-invariance in people do not have the same effect in pigeons. People show rotation costs when recognizing bent-paperclip objects (e.g., Edelman and Bülthoff, 1992), but these costs are reduced when a single diagnostic geometrical volume (“geon”) is added to each object (Tarr et al., 1997). The same results are not observed in pigeons (Spetch et al., 2001), which show decrements in performance as a function of rotational distance regardless of the object components.

On the other hand, pigeons show generalization behavior that is closer to true view invariance as the number of training views is increased (Wasserman et al., 1996; Peissig et al., 1999, 2002). This finding is essentially another manifestation of the category size effect described earlier and can be explained in the same way: that is, as arising from a repetition advantage effect for view-invariant properties, which are repeated often across different views and therefore are frequently paired with the correct responses.

**Figure 8:**
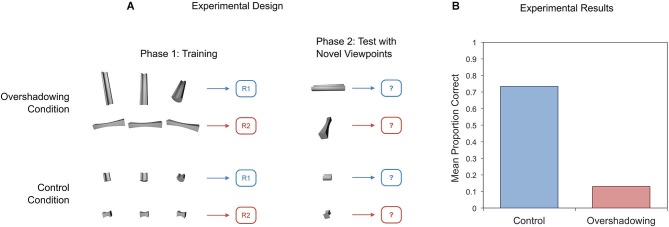
**Schematic diagram of an experiment on overshadowing of view-invariance learning (A) and results from an experiment with pigeons (B)**. Bars in panel **(B)** represent responding to novel views of the training objects presented during Phase 2.

If this explanation of view-invariance learning in pigeons is correct, then it should be possible to arrange conditions in which training with multiple views of an object does not lead to higher invariance, by reducing the advantage of view-invariant properties over other properties during training. Soto et al. (2012) recently tested this hypothesis by training pigeons with object views similar to those shown in Figure 8A. In the training images for the overshadowing condition, across variations in viewpoint, there is a pronounced feature that is not view-invariant and that can perfectly predict object identity: the orientation of the main axis. The repetition of this feature across views should produce something akin to the category overshadowing effect explained earlier and impair view-invariance learning. On the other hand, in the control condition, pigeons are trained with the same views of less elongated objects; this training eliminates the competing non-invariant feature of main-axis orientation, which should result in higher invariance. Figure 8B shows that performance with new views was above chance for the control condition and below chance for the overshadowing condition, just as predicted.

Humans do sometimes show rotational costs in object recognition tasks (Hayward and Tarr, 1997; Tarr et al., 1998), which diminish after training with multiple views (Mash et al., 2007). These findings raise the possibility that view-invariance learning in people might follow similar principles as in pigeons, being driven by prediction errors. A role for error-driven learning has been found in human object categorization (Soto and Wasserman, 2010d) and there is evidence that categorization and identification depend on similar neural representations and computations (e.g., Hung et al., 2005).

This possibility has remained unexplored in the primate literature, which has focused instead on looking for evidence of unsupervised learning of invariant object representations (Cox et al., 2005; Li and DiCarlo, 2008, 2010, 2012). It must be noted that the evidence gathered so far does not rule out a role for reward prediction error in invariance learning. In the monkey experiments carried out by Li and DiCarlo (2008, 2010), for example, animals were rewarded for looking at the presented objects. In similar human experiments (Cox et al., 2005), people were engaged in a task that involved “correct” and “incorrect” responses and learning was not observed when experience was delivered passively (Li and DiCarlo, 2012). Thus, these experiments do involve presentation of explicit and implicit rewards and clearly raise the possibility that learning is driven by reinforcement (Li and DiCarlo, 2010). Although one study (Li and DiCarlo, 2012) reported evidence of unsupervised learning independent of reward magnitude and timing, it did not show that reward is not *necessary* for invariance learning. On the other hand, Yamashita et al. (2010) have provided evidence that reward-based discrimination, and not simple exposure, is necessary for invariance learning at least under some circumstances.

The role of unsupervised learning mechanisms in object recognition by pigeons has also remained unexplored. As our discussion of the primate literature shows, one reason is that it is quite difficult to study unsupervised learning in isolation from the influence of reward, particularly in nonhuman animals. This is an important issue that should be addressed by future research.

### What information is extracted from images by pigeons?

**Figure 9:**
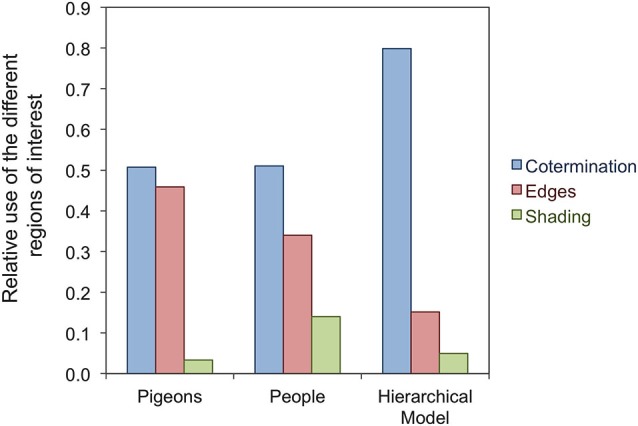
**Relative use of different regions of interest during geon recognition by pigeons, people, and a hierarchical model of object recognition**.

Despite the difference in invariant recognition shown by people and pigeons, there is considerable evidence that the two species rely on similar image information during object recognition tasks (Wasserman and Biederman, 2012). For example, both primates and pigeons seem to extract nonaccidental properties from images of geons and rely heavily on them for recognition (e.g., Biederman and Bar, 1999; Vogels et al., 2001; Kayaert et al., 2005; Gibson et al., 2007; Lazareva et al., 2008). Gibson et al. (2007) trained pigeons and people to discriminate four simple objects, each shown from a single viewpoint. Using the Bubbles technique (Gosselin and Schyns, 2001), it was determined that both species relied more heavily on image properties that are relatively invariant across changes in viewpoint, such as cotermination and other edge properties, than on properties that vary across changes in viewpoint, such as shading. This result is depicted in the leftmost group of bars in Figure 9.

Results such as those shown in Figure 9 do not mean that pigeons rely *only* on view-invariant properties for object recognition. As mentioned earlier, pigeons are sensitive to changes in object viewpoint, size, location, and shading, which means that all of these properties are extracted and used by pigeons during object recognition tasks. The inability of pigeons to show one-shot view invariance is not the result of an inability to extract view-invariant representations. Instead, it is more likely that pigeons extract a rich variety of visual properties from images and can only gradually learn to focus on those that are relevant for a given task through a reinforcement learning mechanism.

**Figure 10:**
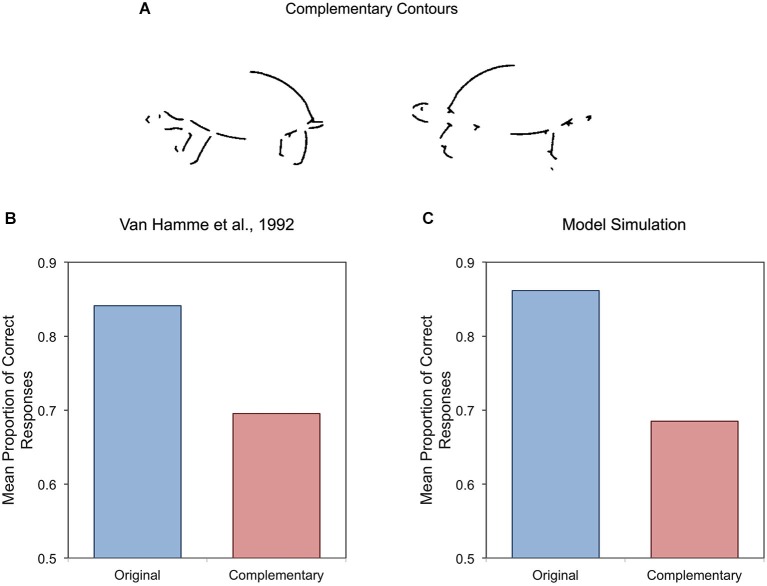
**Examples of the stimuli used by Van Hamme et al. (1992) to study transfer of recognition performance from partial contours to their complementary contours (A), together with the performance of pigeons (B) and a hierarchical model (C) during test**.

Several experiments have found evidence that pigeons represent not only local shape properties, but also the spatial structure of objects (Van Hamme et al., 1992; Wasserman et al., 1993; Kirkpatrick-Steger and Wasserman, 1996; Kirkpatrick-Steger et al., 1998). In one study, Van Hamme et al. (1992) trained pigeons to recognize line drawings of objects, similar to those shown in Figure 10A, in which half of an object’s contour was deleted. This technique allowed the experimenters to train the pigeons with one contour image and to test them with its complement, which shared no local features with the training stimulus. As shown in Figure 10B, pigeons recognized these complementary contours with considerable accuracy, suggesting that their visual system could infer object structure from the partial contours seen during training.

Furthermore, when both shape and spatial relations can be used as cues to solve a recognition task, pigeons rely on both of them and show a trade-off between their reliance on one source of information vs. the other; that is, the more a pigeon relies on shape for recognition, the less it relies on spatial information, and vice-versa (Kirkpatrick-Steger and Wasserman, 1996). Such trade-offs can be explained as another form of overshadowing: when two object properties are equally reliable for identification, they compete for control of performance.

### Feedforward shape processing can explain object recognition in pigeons

Comparative studies have revealed similarities and differences in high-level vision by pigeons and people not only at the behavioral level, as described in the previous section, but also at the neurobiological level. Although primate and avian visual systems are each organized into two main visual pathways, the tectofugal pathway is used for complex visual discrimination tasks in pigeons, whereas the thalamofugal pathway is used for such tasks in primates (Shimizu and Bowers, 1999; Wylie et al., 2009). Still, these pathways show similar functional organization, which has led to the proposal that they might be analogous (Shimizu and Bowers, 1999). For example, the avian tectofugal pathway and its pallial targets are organized into parallel subdivisions in charge of processing motion and shape (Wang et al., 1993; Shimizu and Bowers, 1999; Laverghetta and Shimizu, 2003; Nguyen et al., 2004; Fredes et al., 2010), which is similar to the organization of the primate thalamofugal pathway and its cortical targets (Mishkin et al., 1983; Ungerleider and Haxby, 1994).

Furthermore, there is evidence that one of the main mechanisms thought to be responsible for visual shape processing in the primate thalamofugal pathway is also at work in the avian tectofugal pathway. This mechanism, first proposed by Hubel and Wiesel (1962, 1968), relies on feedforward processing across visual areas that are hierarchically organized in terms of the complexity of the visual information that they represent. Neurons at each level of the system integrate information from neurons at the previous level to build selectivity for shape features of increasing complexity and tolerance to variables such as size and location (for a short review and references, see Soto and Wasserman, 2012c). Li et al. (2007) found that the receptive fields of neurons in the pigeon nucleus isthmus (sensitive to oriented gratings) are constructed by feedforward convergence of receptive fields from neurons in the tectum (which have center-surround organization), as proposed by the hierarchical model of Hubel and Wiesel (1962, 1968). Also in accord with hierarchical processing, there is a large increase in receptive field size from early to later areas in the avian tectofugal pathway (Engelage and Bischof, 1996).

Thus, hierarchical and feedforward processing of shape information–a central mechanism for most current neurocomputational theories of object recognition in primates (e.g., Fukushima, 1980; Perrett and Oram, 1993; Riesenhuber and Poggio, 1999, 2000; Rolls and Milward, 2000; Serre et al., 2007)– might be widespread across vertebrate visual systems. If this is true, then behavioral differences between pigeons and people must be explained by some other mechanism. We Soto and Wasserman (2012c) recently offered a proof of concept for this hypothesis, by showing that a hierarchical model of object recognition in the *primate* ventral stream (a version of the HMAX model described in Serre et al., 2007), coupled with a reinforcement learning model (see Section The Role of Error-driven Reinforcement Learning), can explain much of the available behavioral data in object recognition by *pigeons* reviewed in sub-sections Invariance in Object Recognition by Pigeons and What Information Is Extracted From Images by Pigeons?

The success of this model was surprising for two reasons. First, the model could better explain pigeon behavior than human behavior. Just as pigeons but unlike people, the model’s recognition was strongly affected by changes in viewpoint, size, and shading. In the case of size, the model could even reproduce the logarithmic relation between physical and perceived object size that has been found in pigeons (Peissig et al., 2006). Furthermore, invariant recognition was not fostered by variables that seem to do so for people, such as adding geons to paperclip objects.

Second, although this model uses a “bag of features” to mediate object representation, the results of several simulations showed that such representations can be much richer than one would initially assume. As shown in Figure 10C, the model has no problem reproducing the ability of pigeons to recognize objects from their complementary contours (Van Hamme et al., 1992). This result was originally interpreted as showing that a feature-based representation (such as that proposed by Cerella, 1986)– lacking explicit information about the spatial relations among features–could not explain object recognition in pigeons. This interpretation is only partially correct, because the simulated results suggest that the feature pool in the model can *implicitly* represent information about spatial structure.

The model also reproduces the bias to rely on nonaccidental properties in geon recognition found in people and pigeons (Gibson et al., 2007), as depicted in Figure 9. The model is successful despite the fact that it was not designed to do so, as in the case of other theories of object recognition (structural description theories; see Biederman, 1987). Instead, the bias emerges in the hierarchical model from simple principles of biological visual computing and because the features in the model have been trained through exposure to natural images (see Serre et al., 2007). Coterminations and elongated edges are both quite common in natural images (Geisler et al., 2001) and they could reliably distinguish between the objects used by Gibson et al. (2007).

The success of the hierarchical model in explaining the pigeon behavioral data has no equal in the current literature. Together with the results of neurophysiological studies (Engelage and Bischof, 1996; Li et al., 2007), the success of this model suggests that feedforward and hierarchical processing of visual information play important roles in object recognition by pigeons, as they do in primates.

### The limits of generality: pigeons’ recognition of human faces

Up to this point, we have focused on the mechanisms of visual object recognition that are likely to be shared by pigeons and people. However, the evolutionary lineages of both species diverged more than 300 million years ago; surely, we can expect their visual systems to show important differences due to adaptive specialization.

For example, it is likely that there are specialized mechanisms^1^ of face perception in people and other primates (Pascalis and Kelly, 2009). However, a comparative analysis requires taking into account the fact that face recognition is a complex form of behavior, likely to result from the interaction of many mechanisms, including general processes shared with other species (de Waal and Ferrari, 2010; Shettleworth, 2010). Determining which aspects of human face perception are due to specialized vs. general mechanisms requires comparative research; here, pigeons are becoming a key species to determine the role of general recognition processes (Soto and Wasserman, 2011).

Only a handful of behavioral studies have compared human face recognition by pigeons and people. They have led to a complex pattern of results, suggesting that some properties of face perception in people are likely to be the result of specialized processes, whereas others might result from general processes. Regarding specialized processes, it has been found that, while people and other primates show an advantage in discriminating upright faces over inverted faces, the same advantage is not found in pigeons (Phelps and Roberts, 1994). It is widely believed that faces are perceived in a “holistic” or “configural” way to a larger extent than other objects (for reviews, see Maurer et al., 2002; Richler et al., 2008) and inversion effects have been proposed as a manifestation of holistic face perception (Farah et al., 1995). That is, holistic processing might be a specialized mechanism for face perception in primates.

Surprisingly, other studies have shown similarities in the way people and pigeons process human faces. For example, both species use information near the eyes and chin to discriminate gender and they use information near the mouth to discriminate emotion (Gibson et al., 2005). Also, in both people (e.g., Schweinberger and Soukup, 1998; Fox and Barton, 2007; Ellamil et al., 2008; Fox et al., 2008) and pigeons (Soto and Wasserman, 2011), recognition of emotional expression depends on variations in identity, whereas recognition of identity is relatively independent of variations in emotion. It is possible that the origin of this latter interaction in people is decisional rather than perceptual (Soto et al., 2014), which would make the similarity across species easier to reconcile with the existence of specialized face perception processes in primates.

Overall, these results challenge the common assumption that a specialized human face perception system must underlie all observed aspects of human face recognition, being somehow “encapsulated”, or free from the influence of more general processes. Furthermore, they serve to underscore the fact that the evolution of a face recognition system did not solely involve the specialization of perceptual processes, but also the specialization of the human face as an efficient transmitter of social signals (Smith et al., 2005; Schyns et al., 2009). The human face could have been specialized through evolution to transmit signals that would be easily decoded by existing visual processes. If such visual processes are also present in birds, then the fact that some aspects of face recognition are similar in pigeons and people seems less surprising.

## The evolution of mechanisms of object recognition in vertebrates: a working hypothesis

The ultimate goal of comparative studies of high-level vision is to understand how biological visual systems have evolved mechanisms to solve the challenging computational problems posed by the environment (Soto and Wasserman, 2010a). It is likely that some of the computational problems that are posed by object recognition are present in many environments, leading to the evolution of a core system of processes that are required to solve object recognition tasks across species. Other computational problems may be specific to the environment of one or a few species, leading to the evolution of more specialized processes.

**Figure 11:**
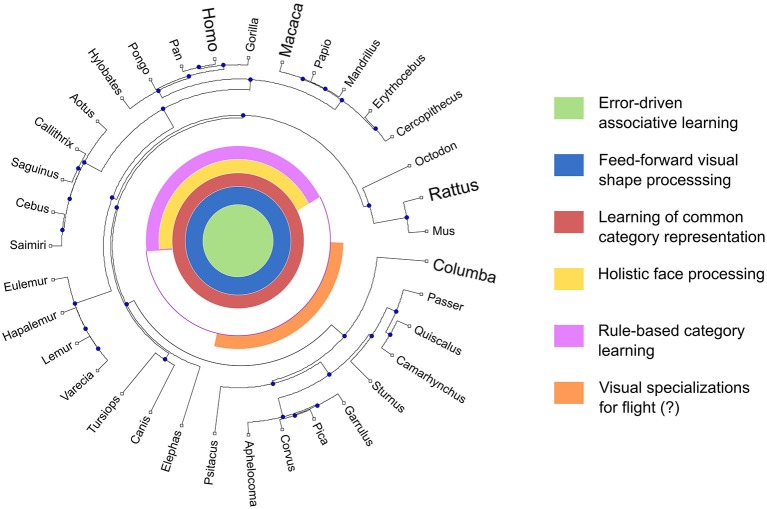
**Diagram summarizing our current working hypothesis regarding the computational mechanisms involved in object recognition across vertebrates**. The outer portion of the diagram consists of a phylogenetic tree, with leafs representing the most commonly studied genera in comparative cognition. The concentric circles at the center represent different hypothesized computational mechanisms. If a line is drawn from a particular leaf to the center of the diagram, then the colors intersected by the line represent those mechanisms hypothesized to be present in that particular genus.

Figure 11 represents our current working hypothesis regarding the evolution of mechanisms of object recognition in birds and mammals. This diagram is a useful way to summarize what is known about the evolution of a complex form of behavior in a large group of animals. The outer part of the diagram consists of a phylogenetic tree, which provides information about the evolutionary relations among species that are being compared. The leaves in this tree include the genera that are most commonly studied in comparative cognition. There is no information about the object recognition abilities of most of these genera; so, they are included simply as a reference. The genera that have been studied to some extent are highlighted: homo (i.e., humans), macaca (macaques) and columba (i.e., pigeons). Rattus (rats) is also highlighted, as recent studies have started to shed light on their object recognition skills (e.g., Zoccolan et al., 2009; Brooks et al., 2013).

The center of the diagram provides information about which species are thought to possess a specific mechanism. Each concentric circle of a different color represents a different hypothetical mechanism. To know which mechanisms are hypothesized in each species, we can draw an imaginary line from that species to the center of the diagram. If the line crosses a colored area in the circle representing a particular mechanism, then this means that the species is thought to possess that specific mechanism. The core system of mechanisms that are shared by many species is shown at the center of the diagram, by circles that are completely colored. More specialized mechanisms are shown toward the periphery.

As illustrated in Figure 11, at least three processes seem to be part of the core system of object categorization in vertebrates: error-driven learning, feedforward processing of visual information, and learning of a common representation for objects in the same category. Of these, there is considerable evidence that error-driven learning is a core mechanism that is present across vertebrates and used in all object categorization tasks. Furthermore, the best candidate structures for implementing this mechanism, the basal ganglia, are homologous across amniote vertebrates, suggesting that this is an evolutionarily conserved mechanism. There is also considerable evidence for feedforward visual processing in primates, but the evidence in other species is less clear. In pigeons, only computational evidence and a couple neurophysiological studies support this hypothesis, so clearly more research is necessary. There is also evidence of learning common representations across all vertebrates, coming from the literature on learned equivalence (see Zentall et al., 2014). Regarding these two latter mechanisms, current neurobiological evidence suggests that they are not implemented in homologous structures across vertebrates, although they are implemented in structures thought to be analogous in birds and primates. These analogous mechanisms could have evolved separately in these different groups, due to similar evolutionary pressures.

Two more specialized mechanisms have been proposed for primates, as shown in Figure 11. We warn that the proposed distribution of these mechanisms across species is highly speculative. Still, the evidence suggests a specialized mechanism for “holistic” face processing in people and other primates, which is not present in birds. It is also likely that birds have evolved specialized mechanisms of visual categorization; for example, flight might have had an important impact on birds’ evolved ability to categorize scenes from different perspectives (Kirkpatrick et al., 2014).

The evolution of a specialized rule-based learning mechanism in primates (and perhaps other mammals) could explain a number of differences found between these species and birds–including many of the differences reviewed here. So, this hypothesis merits more detailed discussion.

There is a growing body of evidence suggesting that at least two learning systems may underlie the categorization abilities of people (e.g., Ashby et al., 1998; Ashby and Ell, 2001; Ashby and Valentin, 2005). One of them is a procedural learning system, believed to be implemented by the circuitry of the basal ganglia and based on slow, error-driven associative learning. The other is a rule-based learning system, believed to be implemented in the PFC and based on hypothesis testing supported by working memory and executive attention. This rule-based system can easily learn category structures in which good performance requires selectively attending to a single dimension, while ignoring other dimensions.

Recent comparative studies (for a review, see Smith et al., 2012a) have suggested a dissociation between these learning systems in people, rhesus monkeys (Smith et al., 2010), and capuchin monkeys (Smith et al., 2012b). On the other hand, neither pigeons (Berg and Grace, 2011; Smith et al., 2011) nor rats (Vermaercke et al., 2014) have shown evidence of such dissociations, even when tested with the same stimuli and similar procedures as people. These results have been interpreted as evidence that the rule-based categorization system is present in primates, but is not found in other mammals and birds.

Assuming that this interpretation is correct, how can we explain the differences between people and pigeons in object recognition tasks? Rule-based learning in people is extremely fast (Smith et al., 2011, 2014) and it generalizes perfectly across irrelevant stimulus dimensions (Casale et al., 2012). Thus, after limited exposure to a specific object, people can selectively attend to those visual dimensions that are important for object identification and ignore those visual dimensions that are irrelevant, such as viewpoint, shading, size, etc. Such learning would require that people separately represent relevant and irrelevant shape dimensions, so that attention can select some dimensions while ignoring others (Demeyer et al., 2007). The results of psychophysical studies agree with this idea: people encode shape information separately from viewpoint information (Stankiewicz, 2002; Blais et al., 2009).

Pigeons, on the other hand, may only slowly learn to select relevant information and ignore irrelevant information through the procedural learning system. That is why pigeons do not show invariant object recognition unless they are trained with variations in irrelevant object dimensions.

This hypothesis also explains why people, but not pigeons, exhibit view-invariant recognition of bent-paperclip objects when a geon has been added to them (Spetch et al., 2001). An ideal observer analysis shows that the task of recognizing objects composed of both bent-paperclips and geons across changes in viewpoint is very difficult, whereas the task of recognizing geons by themselves across changes in viewpoint is very simple (Tjan and Legge, 1998). This analysis suggests that the reason why people show view-invariant recognition of bent-paperclip objects when a geon is added is because they can quickly learn to selectively attend to the geon in order to decrease task difficulty. Pigeons might not be able to show such fast changes of selective attention.

Finally, the hypothesis of a rule-based mechanism present in primates, but not birds, can also explain why many research findings suggest that people and pigeons extract similar information from images, but show performance differences on invariance tests. Similarities could be due to similar visual processing, whereas differences could be due to differences in post-visual processing.

Still, the value of the multiple systems hypothesis depends on how future research is able to eliminate alternative explanations of the comparative results. For example, it is possible that pigeons do posses a rule-based mechanism; but, unlike primates, they do not perceive the dimensions of line width and orientation used by Smith et al. (2011) as separable and thus cannot selectively attend to them. Indeed, some evidence suggests that these dimensions might interact for pigeons (Berg and Grace, 2011; Berg et al., 2014); so, an urgent issue is to determine whether such perceptual interactions do exist using traditional tests of separability adapted to animal research (e.g., Blough, 1988; Soto and Wasserman, 2010c, 2011) or, better still, adapting tests of separability that control for the influence of non-perceptual factors (Ashby and Soto, in press; Soto et al., 2014).

Another possibility is that quantitative differences in visual processes may explain behavioral differences between pigeons and people. Feedforward visual processing gradually increases tolerance to identity-preserving variables across several hierarchically organized layers (see Serre et al., 2007). If the pigeon visual system has a smaller number of layers than the human visual system, then we could expect pigeons to show object recognition that is more sensitive to changes in size, rotation, etc.

Although this is an interesting possibility, it cannot explain why primates, but not pigeons, seem to use two different strategies to categorize artificial stimuli varying along dimensions that are not identity-preserving in natural objects (width and orientation of lines, see Smith et al., 2012a). Furthermore, this hypothesis cannot explain why people show invariant recognition in some behavioral studies after experience with a single image of a novel object. Such behavioral invariance (in contrast to the invariance shown by neurons), requires a readout mechanism that is able to ignore variations along identity-preserving variables (Goris and Op de Beeck, 2009, 2010). The availability of a rule-based readout mechanism in people would allow one to explain why humans can show invariant recognition after experience with a single image of an object. The absence of such a readout mechanism in pigeons would explain why this species does not show this behavior.

If the hypothesis of multiple learning systems turns out to be correct, then future research will be required to determine exactly which aspects of the rule-based system are specialized in primates. As indicated earlier, the NCL is an area of the pigeon brain that seems to support the same executive functions as the primate PFC (Güntürkün, 2005). Thus, it is likely that some of the mechanisms involved in the rule-based system are available to pigeons, and the main difference from people is either merely quantitative or restricted to a few of the processes involved in rule learning.

One possibility is that pigeons do not deploy selective attention in the same way as primates (Smith et al., 2012a) or that they do not perceive any visual dimensions independently, but process all stimuli holistically (Berg et al., 2014). These ideas are in line with studies of compound generalization in pigeon associative learning, which suggest that pigeons process visual stimulus compounds as configurations rather than as the simple sum of their component elements (e.g., Rescorla and Coldwell, 1995; Aydin and Pearce, 1997), whereas people show much more elemental processing in analogous tests (e.g., Collins and Shanks, 2006; Soto et al., 2009). Although pigeons might deploy some forms of dimensional attention during categorization tasks (Mackintosh and Little, 1969; but see Hall and Channell, 1985; Castro and Wasserman, 2014), perhaps the fast switching of dimensional attention that is required for testing hypotheses about category rules is unique to primates (for more on selective attention in pigeons, see Zentall, 2012; Vyazovska et al., 2014).

Although the rule-based system is also thought to require holding hypotheses about possible rules in working memory, it has been shown that neurons in the pigeon NCL–the area of the avian brain also thought to be involved in learning of abstract category representations (Kirsch et al., 2009)—have similar working memory functions as neurons in the primate PFC (Diekamp et al., 2002; Rose and Colombo, 2005). This fact makes it unlikely that working memory is the critical component of the rule-based system that is absent in pigeons.

## The neurobiological mechanisms of object recognition: what we *can* learn from pigeons

The neuroscience community has focused almost exclusively on nonhuman primates for studying the neurobiology of visual cognition, perhaps due to their evolutionary proximity to humans. From a truly comparative standpoint, however, other animals are just as useful as nonhuman primates for the study of the core processes involved in visual object recognition. Using pigeons as an animal model for the study of object recognition offers many advantages. The most important advantage, as demonstrated by the present review of the literature, is that we know far more about pigeons’ object recognition abilities than about those of any other species, excluding people and rhesus macaques. Furthermore, comparative data are available for most human results in the pigeon literature, so we have a good idea as to just what is similar and different in people and pigeons; such parallel data sets help us understand the limits of our generalizations from the animal model to humans. Finally, behavioral and neurobiological evidence suggests that birds possess highly advanced visual systems, comparable to those of primates in their level of sophistication (Shimizu and Bowers, 1999; Cook, 2001; Husband and Shimizu, 2001; Wasserman and Zentall, 2006).

Given these advantages, it is rather puzzling that pigeons are not being used more widely as a model for the neurobiological basis of object recognition (and other forms of high-level vision). Worse still, neuroscientists studying object recognition in primates have thus far ignored the behavioral and neurobiological literature on pigeons as a source of information for their own research. This omission suggests an implicit belief that this literature is useless for understanding human vision, perhaps due to the evolutionary distance between pigeons and people. We believe that this position comes both from the unfortunate, but popular misconception about the pigeon brain and from the failure to adopt a truly comparative approach in the study of visual and cognitive neuroscience.

The reluctance to accept the idea that anything about the primate brain can be learned from the study of the avian brain might have its origins in the old terminology used to describe bird brains, which suggested that these consist entirely of basal ganglia (Colombo and Scarf, 2012). This perspective is now outdated (Reiner et al., 2004, 2005; Jarvis et al., 2005), as there is considerable evidence that an important proportion of the avian brain consists of pallial areas, many of them homologous to cortical areas in mammals.

Current thinking in comparative psychology recognizes that most forms of complex behavior are the result of many underlying processes, some of them specialized in a single species, others shared across many species, and most somewhere in between these extremes (de Waal and Ferrari, 2010; Shettleworth, 2010; Soto and Wasserman, 2012c). No species will provide a perfect animal model of human behavior. For example, comparative studies have found differences between the human brain and that of other primates–including great apes–across all studied levels of organization, from genes to the size and connectivity of large areas (Preuss, 2011).

All of this work suggests that the only way to appropriately use animal models is by understanding what is shared and what is not between people and each specific model animal. Unfortunately, a much more common approach is to choose a model animal based on face validity and to glibly assume that the mechanisms underlying behavior in the model animal are similar to those in people. The belief that a species that is closer to people in the phylogenetic tree must provide a better model for any cognitive process is one manifestation of such reliance on face validity. Underlying this idea is the (clearly incorrect) assumption that the rate of evolutionary change is fixed across traits, environments, and species. From a truly comparative perspective, researchers should avoid relying on face validity to choose the species that they study. Instead, they should rely on the results of comparative studies–including behavioral research. In precisely this respect, the pigeon offers many manifest advantages.

We propose that pigeons can provide an excellent animal model for the study of the core processes involved in visual object recognition. Only in the study of specialized processes may other models be proven to afford a better alternative. In those cases, researchers should seek strong behavioral evidence regarding the computational mechanisms involved, just as has been done in pigeons over the last 50 years. After such research is performed, we would be in a better position to determine exactly what we are studying when we investigate object recognition in such species. Fortunately, we do not need to take another 50 years in order to reach a good understanding of the mechanisms of object recognition in rats, cats, and other mammals, as we can learn from the successes and failures of the pigeon research.

We have shown here that the behavioral study of object recognition in pigeons has yielded important insights into the general computational mechanisms used by vertebrates to solve this vital visual task and into the evolution of these mechanisms. Similarly, we believe that much will be learned about the neurobiology of object recognition from the study of the “simple” brains of pigeons.

## Conflict of interest statement

The authors declare that the research was conducted in the absence of any commercial or financial relationships that could be construed as a potential conflict of interest.

## Footnotes

^1^Note that *specialized* and *general* are used here to refer to the distribution of a cognitive mechanism across species, with specialized referring to a mechanism that can be found in only a few species and general referring to a mechanism that can be found across a variety of species. The distribution of a mechanism across species should in turn depend on whether the computational problem solved by such mechanism is widespread across environments (see Soto and Wasserman, 2012c). Importantly, how a mechanism is distributed across species is different from the issue of whether such mechanism is domain-general or domain-specific. Thus, when we propose that any complex ability is likely to be influenced by specialized processes, we mean processes that are only present in one or a few species (e.g., language), not processes that are domain-specific.
